# PAPE (Prefractionation-Assisted Phosphoprotein Enrichment): A Novel Approach for Phosphoproteomic Analysis of Green Tissues from Plants

**DOI:** 10.3390/proteomes1030254

**Published:** 2013-12-05

**Authors:** Ines Lassowskat, Kai Naumann, Justin Lee, Dierk Scheel

**Affiliations:** 1 Leibniz Institute of Plant Biochemistry, Weinberg 3, Halle/Saale, D-06120, Germany; E-Mails: Ines.Lassowskat@ipb-halle.de (I.L.); dscheel@ipb-halle.de (D.S.); 2 Riboxx GmbH, Meissner Str. 191, Radebeul, D-01445, Germany; E-Mail: Kai.Naumann@riboxx.com

**Keywords:** phosphoproteomics, LC-MS, *Arabidopsis thaliana*, phosphoprotein enrichment

## Abstract

Phosphorylation is an important post-translational protein modification with regulatory roles in diverse cellular signaling pathways. Despite recent advances in mass spectrometry, the detection of phosphoproteins involved in signaling is still challenging, as protein phosphorylation is typically transient and/or occurs at low levels. In green plant tissues, the presence of highly abundant proteins, such as the subunits of the RuBisCO complex, further complicates phosphoprotein analysis. Here, we describe a simple, but powerful, method, which we named prefractionation-assisted phosphoprotein enrichment (PAPE), to increase the yield of phosphoproteins from *Arabidopsis thaliana* leaf material. The first step, a prefractionation via ammonium sulfate precipitation, not only depleted RuBisCO almost completely, but, serendipitously, also served as an efficient phosphoprotein enrichment step. When coupled with a subsequent metal oxide affinity chromatography (MOAC) step, the phosphoprotein content was highly enriched. The reproducibility and efficiency of phosphoprotein enrichment was verified by phospho-specific staining and, further, by mass spectrometry, where it could be shown that the final PAPE fraction contained a significant number of known and additionally novel (potential) phosphoproteins. Hence, this facile two-step procedure is a good prerequisite to probe the phosphoproteome and gain deeper insight into plant phosphorylation-based signaling events.

## 1. Introduction

The completion of the genome sequencing in 2000 [1] has further propelled *Arabidopsis thaliana* into one of the most well-established model organisms to study plant molecular biology/biochemistry [2]. *Arabidopsis* is used for a wide range of “OMICS” analysis concerning genes (genomics; [3,4]), proteins (proteomics; [5,6,7]) and metabolites (metabolomics, [8]). One sub-topic of proteomics, rising in the last few years, is the field of phosphoproteomics [9]. The strong interest originates from the importance of protein phosphorylation for the biochemistry of all organisms, especially in regulating cellular processes, ranging from cell differentiation, development, cell cycle control, metabolism and signal transduction [10,11,12]. Probably 30% of all proteins are phosphorylated at any given time and state [13], indicating the immense dimension of the phosphoproteome. Beside its different roles in the regulation of protein synthesis, gene expression and apoptosis, phosphorylation events exhibit a pivotal role in defense responses [14]. An example is the activation of mitogen-activated protein kinase (MAPK)-mediated phosphorylation signaling cascades upon stress or other environmental signals [15,16,17]. The corresponding downstream targets of such a cascade are, to a great extent, unknown. For further understanding of defense mechanisms in plants, more knowledge about signaling cascades is of high significance. Therefore, a fully developed strategy for phosphoprotein/peptide enrichment is necessary.

Unfortunately, plant phosphoproteomics using leaf material can be a challenging task. Not only the presence of highly abundant proteins, like RuBisCO, but also the low levels of phosphorylated signaling proteins limit their visualization and detection on PAGE-gels. Even highly advanced mass spectrometry is often unable to recover large numbers of phosphopeptides in complex samples. Common methods frequently describe the enrichment of phosphopeptides prior to measurement to overcome this challenge. Most methods use metal ions for the binding of phosphopeptides, for instance, chelated metal ions (immobilized metal affinity chromatography IMAC); [18,19]) or metal oxides (metal oxide affinity chromatography (MOAC); [20]). Other methods describe the use of multi-step procedures, in which a first enrichment of phosphoproteins should assist the subsequent phosphopeptide enrichment [21]. Nevertheless, one disadvantage of such an approach is that not all phosphopeptides are efficiently captured, and also, information concerning the non-phosphorylated peptides is lost, which may impede target identification, for instance, in the cases of highly similar proteins of multigene families [22]. Other approaches first remove highly abundant proteins that might interfere with the applied phospho-enrichment matrix. In plants, this means the reduction or depletion of RuBisCO prior to phosphoprotein enrichment [23,24]. A popular way to accomplish the fractionation of proteins is salting out with chemicals. Polyethylene-glycol (PEG)-based fractionation, for instance, has been successfully employed for improved proteome coverage, leading to the detection of differentially-expressed proteins of low abundance [25,26]. However, since the remaining PEG can interfere in MS analysis, we tested here another commonly used fractionation, namely, ammonium sulfate (AS) precipitation. In previous work done in our laboratory, it could be shown that a reduction of the RuBisCO content via AS precipitation had a positive effect on the preparation of 2D-PAGE, as well as the enrichment of phosphoproteins [27]. As a further improvement for phosphoprotein analysis, we now incorporated the metal oxide affinity chromatography (MOAC) method [20] to the AS-based RuBisCO removal step, which, by itself, already acts as prefractionation/enrichment of phosphoproteins. This led to a facile, but efficient, phosphoproteome analysis procedure, which we termed prefractionation-assisted phosphoprotein enrichment (PAPE). 

## 2. Experimental

### 2.1. Plant Growth

*Arabidopsis thaliana* (Col-0) seeds were grown in soil. After two days of stratification at 4 °C, the plants were maintained under short-day conditions (8 h, 200 µE, 23 °C) for six weeks prior to protein extraction. 

### 2.2. Protein Extraction

Leaf material was ground to a fine powder in liquid nitrogen and mixed vigorously with 3 volume of extraction buffer (100 mM HEPES-KOH, pH 7.5; 5% glycerol; 5 mM EDTA; with freshly added 0.1% mercaptoethanol, 1% proteinase and phosphatase inhibitors 2 + 3 from Sigma Aldrich, Taufkirchen, Germany) for 20 min (4 °C). The suspension was centrifuged at 3,220 ×*g* for 15 min. The supernatant was filtered [0.45 µm cellulose mixed ester (CME) filter, Roth, Karlsruhe, Germany]. 

### 2.3. Precipitation of Protein Extract

(a) *Fractionation of proteins.* Ammonium sulfate was added to a final concentration of 40% saturation and incubated for half an hour (4 °C). After centrifugation (3,220 × *g*, 15 min, 4 °C), the pellet was washed twice with wash solution (80% acetone, 20% Tris-HCl (50 mM, pH 7.5); −20 °C) and once with ice-cold acetone. 

(b) *Precipitation of total proteins.* An equal volume of Tris-EDTA-buffered phenol (Roth) was added, mixed vigorously for 1 min and incubated for 5 min at 4 °C. After centrifugation (3,220 × *g*, 15 min, 4 °C), the phenolic phase was transferred and re-extracted twice with 1 volume of re-extraction buffer (100 mM Tris-HCl, pH 8.4, 20 mM KCl, 10 mM EDTA and freshly added 0.4% (v/v) β-mercaptoethanol). The final phenolic phase was mixed with 5 volume of precipitation solution (100 mM ammonium acetate in methanol; −20 °C), incubated over night at −20 °C, and the proteins pelleted by centrifugation (3,220 ×*g*, 15 min, 4 °C). The pellet was washed once with precipitation solution and twice with wash solution. The pellets (from a and b) were air dried and solubilized in LysShot buffer (8 M urea, 50 mM Tris, pH 8.5) or in MOAC incubation buffer (30 mM MES, 20 mM imidazole, 200 mM aspartate, 200 mM glutamate, 0.25% 3-[(3-cholamidopropyl)dimethylammonio]-1-propanesulfonate (CHAPS), 8 M urea, pH 6.1) for samples to be processed by the MOAC step. 

### 2.4. Phosphoprotein Enrichment (MOAC)

Forty milligrams of Al(OH)_3_ matrix (Sigma-Aldrich) were equilibrated with 1.8 mL of incubation buffer (see Section 2.3). A 1.5-mL sample with a protein concentration of 0.5 µg/µL was loaded and incubated by rotating for 30 min (4 °C). After incubation, the matrix was washed four times with incubation buffer. The proteins were eluted twice (800 µL and 400 µL) with tetrapotassium pyrophosphate (TKPP) buffer (8 M urea, 100 mM TKPP, pH 9.0) for 45 min at room temperature [20], and centrifuged (18,514 ×*g*, 2 min, 15 °C) to pellet the Al(OH)_3_ matrix. The pooled eluates were centrifuged twice (18,514 ×*g*, 2 min, 15 °C), to pellet any remaining matrix, and, subsequently, concentrated with centricon filter devices (3 kDa cut-off; Millipore, Bilterica, MA, USA). Proteins were precipitated with a 2D-CleanUp kit (GE Healthcare, Hercules, CA, USA), according to the manufacturer’s instructions, and solubilized in LysShot (see Section 2.3). 

### 2.5. SDS-PAGE and Phosphoprotein Staining

Protein concentration was determined by a 2-D Quant Kit (GE Healthcare). SDS-PAGE was carried out according to [28] by using Precast Gels (Criterion Tris-HCl 12.5%; Biorad, Munich, Germany). Ten micrograms of each sample in loading buffer (0.313 M Tris-HCl, pH 6.8, 50% glycerol, 10% SDS, 0.05% (w/v) bromophenol blue, 0.5 M dithiothreitol (DTT) were heated for 5 min at 95 °C and cooled to room temperature prior to loading. Peppermint Stick^TM^ Phosphoprotein Molecular Weight Standard (Life technologies, Darmstadt, Germany) was used as the molecular weight marker. Pro-Q Diamond (Life technologies) staining was carried out according to a modified protocol [29]. Fluorescent images were obtained using the Typhoon scanner (GE Healthcare) with the settings: 532 nm excitation, 580 nm band pass emission filter and the photo multiplier tube at 550. ImageJ software (National Institute of Health, Bethesda, MD, USA) was used for false color representation. Total protein was visualized with Novex^®^ Colloidal Blue Staining Kit (Life Technologies). 

### 2.6. In-Solution Digestion

Protein concentration was determined by a 2-D Quant Kit (GE Healthcare), and the proteins (in LysShot) were reduced with 200 mM DTT (in 100 mM Tris, pH 7.8) for 1 h and, subsequently, alkylated with 200 mM iodoacetamide (in 100 mM Tris, pH 7.8) for 1 h at room temperature. The solution was diluted to 0.5 M urea with 50 mM NH_4_HCO_3_ (pH 8) and digested overnight with sequencing grade trypsin (Promega, Mannheim, Germany) at a ratio of 1:50 at 37 °C. Peptides were desalted on C18 tips or columns (Protea, Morgantown, WV, USA; Thermo, Bonn, Germany) and reconstituted in solution containing 5% acetonitrile (ACN) and0.1% trifluoroacetic acid (TFA). 

### 2.7. Mass Spectrometry

Tryptic digests were analyzed with an LC-MS system consisting of a nano-LC (Easy-nLC II, Thermo Fisher Scientific, Bremen, Germany) coupled to a hybrid-Fourier Transform (FT)-mass spectrometer [Linear Trap Quadrupole (LTQ) Orbitrap Velos, Thermo Fisher Scientific]. Peptide separations were performed on a C18 column (EASY column; 10 cm, ID 75 µm, particle diameter: 3 µm) at a flow rate of 300 nL/min and a linear gradient of 5% to 40% B in 150 min (A: 0.1% formic acid in water, B: 0.1% formic acid in ACN). A voltage of +1.9 kV was applied to electrospray peptide ions. A capillary temperature of 275 °C for peptide transfer and a lock mass of 445.120024 *m/z* were used. Precursor mass scanning was performed from 400 to 1,850 *m/z* in the Orbitrap with a resolution of 30,000, and the 20 most intense precursor ions were selected for subsequent collision-induced dissociation (CID) fragmentation in the linear quadrupole mass analyzer (LTQ). Singly-charged ions were rejected from fragmentation. Dynamic exclusion was enabled (repeat count: 1; repeat duration: 20 s; exclusion list size: 500; exclusion duration: 30 s). 

### 2.8. Spectral Data Analysis

MS raw data were searched against an *A. thaliana* protein database based on The Arabidopsis Information Resource (TAIR) 10 with the Proteome Discoverer 1.3 using an in-house Mascot server (precursor mass tolerance: 7 ppm; fragment mass tolerance: 0.8 Da; missed cleavages: 2). Carbamidomethylation of cysteine was set as a static modification. Variable modifications were oxidation (Methionine), acetylation (protein *N*-terminus), deamidation (Asparagine/Glutamine) and phosphorylation (Serine/Threonine). Further data evaluation was carried out with the Scaffold software (Version 3.3, Proteome Software Inc., Portland, OR, USA), Proteome Discoverer 1.3 with phosphoRS 1.0 (Thermo Fisher Scientific) and DanteR [30] for total protein content. Phosphopeptides were identified with the Proteome Discoverer 1.3 software, which includes the phosphoRS 1.0 algorithm (Thermo Fisher Scientific) for phospho-site mapping. A false discovery rate (FDR) was calculated by searching a “decoy” database containing all the target database sequences in reverse order. Peptide-spectrum match (PSM) was set at a q-value <0.05 (*i.e*., a corrected significance threshold employing the Benjamini-Hochberg FDR procedure to control for a family-wise error rate). Protein grouping was enabled. Gene ontology (GO) annotation was achieved with the tool on TAIR [31]. The mass spectrometry proteomics data have been deposited to the ProteomeXchange Consortium (http://proteomecentral.proteomexchange.org) via the PRIDE (PRoteomics IDEntifications) partner repository [32] with the dataset identifier PXD000421. 

## 3. Results and Discussion

### 3.1. Prefractionation of Arabidopsis Leaf Proteins

**Figure 1:**
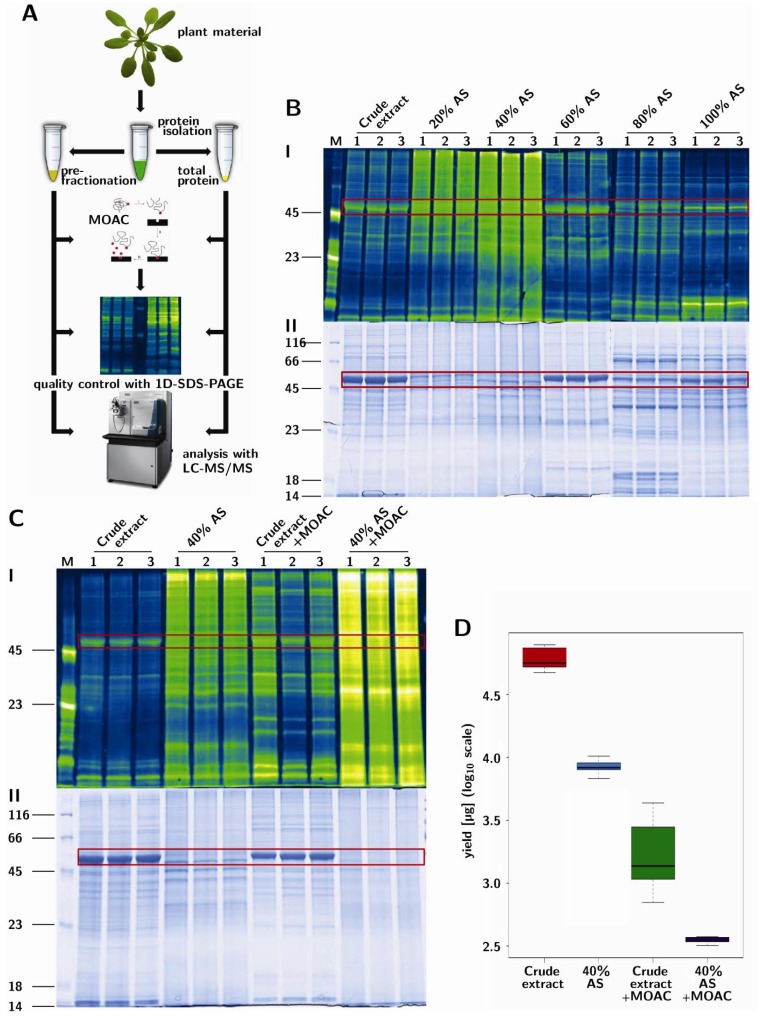
Prefractionation-assisted phosphoprotein enrichment (PAPE). (**A**) Experimental setup. *Arabidopsis* soluble leaf proteins were either extracted with phenol to obtain the crude extract (total protein) or pre-fractionated by 40% ammonium sulfate precipitation. Subsequently, phosphoproteins were enriched using metal oxide affinity chromatography (MOAC). Quality control was assessed with SDS-PAGE and phospho-specific Pro-Q Diamond staining. Mass spectrometry analysis was performed with on-line nano-LC (Easy-nLC II) FT-mass spectrometry (LTQ Orbitrap Velos); (**B**) SDS-PAGE showing the crude extract and stepwise ammonium sulfate (AS) fractionations. Each step was performed three times (lanes labeled 1, 2 and 3). Visualization of proteins was achieved with (I) Pro-Q Diamond phosphoprotein staining in false-color representation and (II) colloidal coomassie staining. Protein molecular weights are indicated on the left-hand margin. (**C**) SDS-PAGE of different extraction and phosphoprotein enrichment steps, with visualization of the proteins as described above. Boxed areas mark the position of the large subunit of RuBisCO. (**D**) Boxplot depiction of protein yield (log10 microgram of proteins) from 25 g of leaf material (n = 6).

A stepwise fractionation with ammonium sulfate (AS) was used to salt out proteins in solution. This was done with 20% AS increment steps, while pelleting precipitated proteins by centrifugation after every step. The molecular weight distribution of proteins in the AS steps was determined with 1D-PAGE (Figure 1B). The large subunit of RuBisCO (boxed, Figure 1B, lower panel), which is one of the most abundant proteins in the non-fractionated sample (crude extract), is predominantly located in the fractionation steps using more than 40% AS. Serendipitously, the fractions produced with 20% and 40% AS (with little or no apparent RuBisCO content) also contained the most phosphoproteins, as evidenced by phospho-specific Pro-Q Diamond staining (Figure 1B, upper panel). In contrast, the samples from the 60%–100% AS fractionation steps showed only very low levels of phosphoproteins. Therefore, the sample precipitated with 40% AS is an excellent source for subsequent phosphoprotein enrichment and represents the first step of the method described below, which we called prefractionation-assisted phosphoprotein enrichment (PAPE).

### 3.2. PAPE: Prefractionation-Assisted Phosphoprotein Enrichment

Crude extract and the 40% AS fraction (40% AS) were subjected to phosphoprotein enrichment with metal oxide affinity chromatography (MOAC) [20] (Figure 1A) with minor modifications, as described in the Experimental section. To evaluate the reproducibility and efficiency of the PAPE procedure (a combination of AS precipitation followed by MOAC), the total extract, the 40% AS fraction and the corresponding MOAC-enriched fractions were each prepared three times, separated on a 1D-PAGE and visualized by coomassie brilliant blue and Pro-Q Diamond phosphospecific staining (Figure 1C). As observed in the stepwise fractionation, the non-fractionated samples had the least visible phosphoprotein content (crude extract). While a faint enrichment effect could be achieved via MOAC (crude extract + MOAC), the prefractionation (40% AS) already had a high phosphoprotein content, which was dramatically increased in combination with the additional MOAC phosphoprotein enrichment step (40% AS + MOAC, Figure 1C). We will hereafter refer to this “40% AS + MOAC” fraction as the PAPE fraction.

Notably, on the basis of the prepared replicates shown here, the “MOAC-only” method (crude extract + MOAC) had a larger variability in phosphoprotein enrichment compared to the other procedures (Figure 1C, upper panel; see, also, the standard deviation of the box plot in Figure 1D). Moreover, the “MOAC-only” fractions contained substantial amounts of RuBisCO (Figure 1C, lower panel), which may be a hindrance in subsequent mass spectrometry-based detection of less abundant proteins [33]. The PAPE fraction showed no distinct bands, but a uniform distribution across all molecular masses in the coomassie, as well as in the phosphospecific stain. Hence, the combination of 40% AS fractionation served both to remove RuBisCO and to enrich for phosphoproteins. The final protein yield by the PAPE procedure is about 0.6% of the total crude extract (Figure 1D); assuming all these are phosphoproteins, this is in agreement with the total phosphoprotein amount expected. 

### 3.3. Reproducibility and Robustness of PAPE on the Basis of Mass Spectrometry Analysis

**Figure 2:**
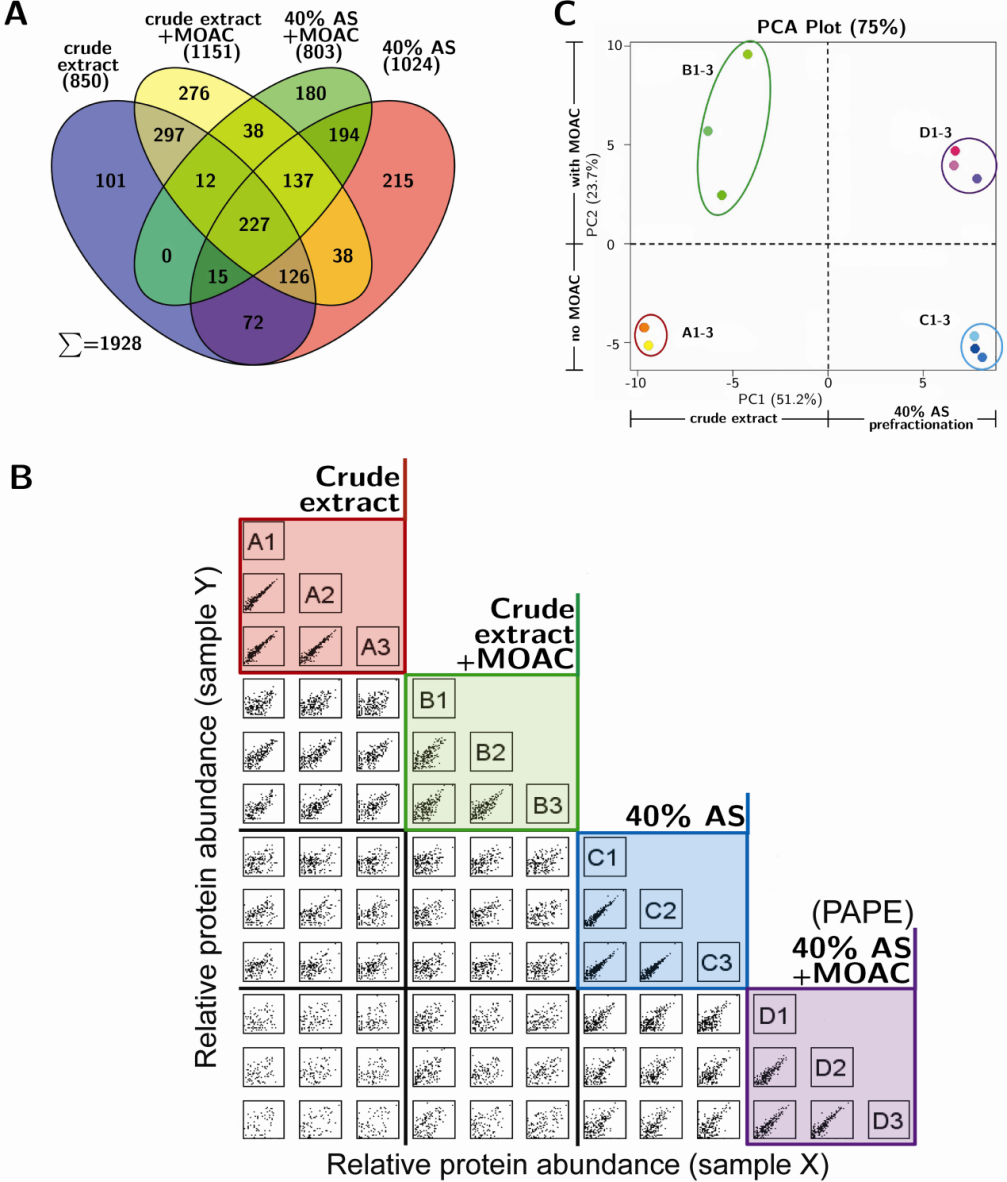
Mass spectrometry analysis of proteins from the fractionation steps of the PAPE procedure. (**A**) Flower plot showing the qualitative differences in the protein composition of the various PAPE fractions. The numbers are the total number of proteins identified from three experiments, with each sample being measured twice; (**B**) Variability and reproducibility of the PAPE procedure. Each small square represents a scatter plot of protein abundance (quantitative values based on spectral counting, SCAFFOLD; DanteR [30]) of the intersecting samples from the various fractionation steps. The letters A–D denote crude extract, crude extract + MOAC, 40% AS and 40% AS + MOAC, respectively, and the numbers 1–3 correspond to the three replicate experiments for each fractionation step. Note the strong positive correlation within the three replicate experiments of each fractionation step (colored boxes); (**C**) Principal Component Analysis (PCA) plot. The dashed lines divide the plot into sectors along the weight of the principal components separating with/without prefractionation and MOAC phosphoprotein enrichment steps, respectively.

In addition to the coomassie and phosphostain gel-based analysis, mass spectrometry may provide a more qualitative estimation of the PAPE efficiency. Tryptic peptides derived from two micrograms of proteins from each of the three replicates of the four fractionation steps (*i.e*., crude extract, 40% AS fraction and the corresponding MOAC-treated samples of these two fractions) were measured with shotgun LC-MS. Each sample was measured in two LC-MS runs and the proteins identified for each fractionation step pooled from both runs. This led to the identification of 850, 1,024, 1,151 and 803 proteins from the crude extract, the 40% AS fraction and their corresponding MOAC-treated samples, respectively (SCAFFOLD Software parameters: minimum protein probability 99.0%/minimum number of peptides 2/minimum peptide probability 90%). These represent a total of 1,928 unique proteins, and the distribution in the four fractionation steps is illustrated in Figure 2A. The identities of these 1,928 proteins are listed in Supplemental Table S1. 

The qualitative protein composition varied greatly between fractions. In fact, of the total 1,928 proteins identified, only 227 proteins were common to all fractions, thus suggesting that the fractions contain different subsets of proteins (Figure 2A). The overlap between the crude extract and the 40% AS fraction was 440 proteins (~50% of the crude extract), indicating that 40% AS precipitated a subset of the total proteins, as is expected when considering the wide range of protein solubility in aqueous solvents [34]. Surprisingly, the overlap between the crude extract and the MOAC-enriched fraction revealed 662 proteins, which represents ~78% of the crude extract. Since it is unlikely that 78% of the identified proteins in the crude extract are phosphoproteins, it hints at substantial unspecific binding to the metal oxide. For instance, this might be due to binding to the negative charges provided by carboxylate moieties within proteins [35], which can exacerbate the binding of phosphoproteins in complex protein mixtures. These problems of the MOAC step in capturing non-phosphorylated targets is partially alleviated by the PAPE procedure described here, since the AS-prefractionation is already enriched for phosphoproteins (see Figure 1C). Therefore, the PAPE procedure is clearly advantageous compared to using only MOAC in phosphoproteomics. 

The high technical reproducibility of each fractionation step can be seen in the positive linear relationship in the scatter plot of the quantitative value (based on spectral counting, SCAFFOLD; DanteR [30]) of each identified protein between the replicate experiments (see the colored boxes in Figure 2B). Notably, the tighter clustering of the replicates from the PAPE procedure when compared to the MOAC samples (purple box *versus* green box, respectively; Figure 2B), as well as the grouping within a Principal Component Analysis (Figure 2C) supports the robustness of the PAPE method over the MOAC method. 

### 3.4. Validation of Phosphoprotein Enrichment by the PAPE Procedure

**Figure 3:**
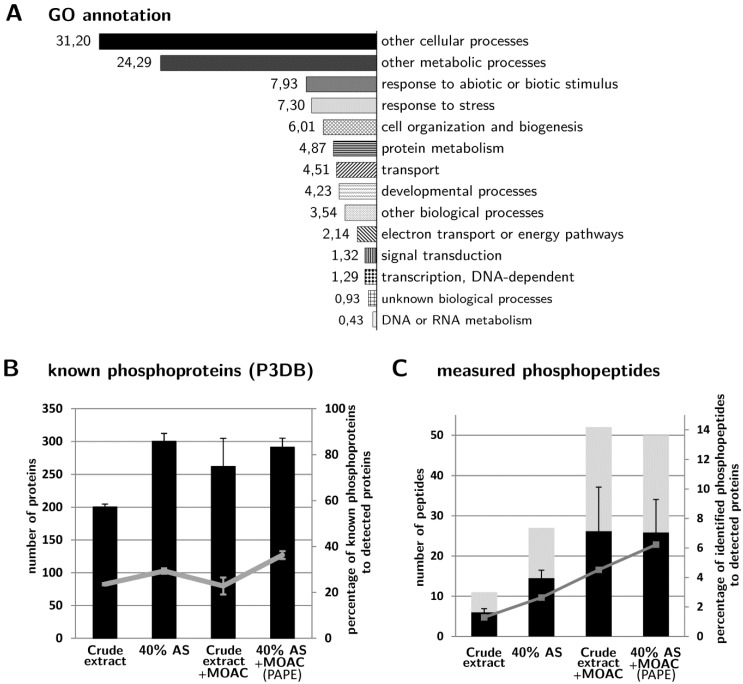
Changes of the protein/phosphoprotein composition. (**A**) Gene ontology functional categorization (based on the The Arabidopsis Information Resource (TAIR) gene ontology (GO) web-tool) of the proteins detected with the PAPE procedure; (**B**) The number of identified proteins in the various fractionation steps that are annotated as known phosphoproteins in the P3DB database. The grey line represents the percentage of identified known phosphoproteins to the total number of identified proteins in each fraction (see Figure 2A); (**C**) The number of phosphopeptides identified in the various fractionation steps. (Only high-confidence phosphopeptides with a phosphorylation site probability (pRS) score >30 are considered; for a full list, see Table S2). Each experiment was performed three times and measured twice. Black bars are the average number of phosphopeptides (+/−standard deviation) detected in each fraction, while grey bars depict the total number of non-identical phosphopeptides identified from all replicates. The grey line depicts the percentage of identified phosphopeptides to the total number of identified proteins in each fraction.

Figure 1C demonstrated that the PAPE procedure precipitated and enriched phosphoproteins. To further support this Pro-Q Diamond phosphostain evidence (Figure 1C), we determined if there was indeed an increase in the identification of known phosphoproteins from the fractionation steps. Using P3DB, a curated plant phosphoprotein database that contains only experimentally verified high quality entries, we found that the 40% AS, MOAC and PAPE fractions contain significantly higher numbers of known phosphoproteins than the crude extract (Figure 3B). Since the number of identified proteins varied between fractions, we also calculated the identified known phosphoproteins as a percentage of all identified proteins within each fraction (grey line in Figure 3B) in order to circumvent any misrepresentation. This demonstrated that the PAPE fraction had proportionally more known phosphoproteins than the MOAC fraction (36% and 23%, respectively), thus suggesting the improvement of the PAPE procedure over MOAC alone to enrich phosphoproteins. Gene ontology (GO) annotation of the proteins identified in the PAPE fraction showed an enrichment of proteins involved in response to abiotic and biotic stimuli and to stress (Figure 3A). Since protein phosphorylation regulates many of these processes, it supports the effectiveness of PAPE to enrich lowly abundant phosphorylated proteins that are also involved in cellular signaling.

**Figure 4:**
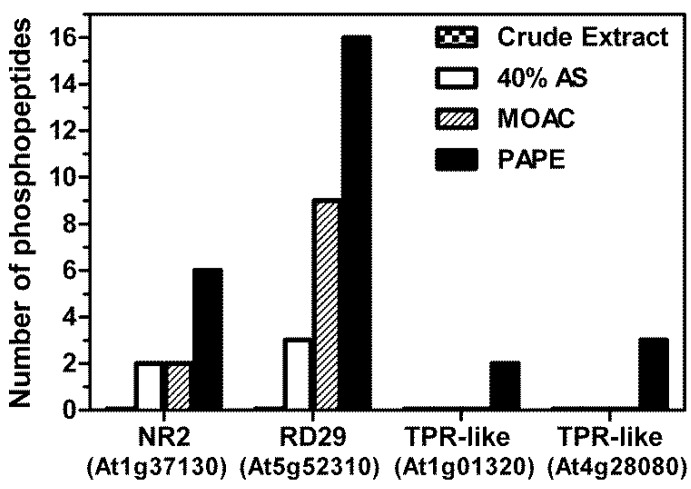
Examples of the increased detection of phosphopeptides associated with a particular protein in the PAPE fraction. A progressively increasing number of phosphopeptide detections is seen for the listed proteins from the crude to the PAPE fraction. (Abbreviations: NR2, nitrate reductase 2; RD29, desiccation-responsive protein 29, which is also known as low-temperature-responsive protein 78; TPR-like, proteins from the tetratricopeptide repeat superfamily).

Correspondingly, we identified more phosphopeptides in the 40% AS, MOAC and PAPE fractions than in the crude extract (Figure 3C). In particular, when represented as the percentage of phosphopeptides relative to the total number of proteins identified in each fraction, more phosphopeptides were recorded in the PAPE than the MOAC fraction (6.2% and 4.5%, respectively). Interestingly, many of the phosphopeptides detected in the PAPE fractions were not listed in the P3DB [36,37] and PhosPhAt 3.0 [38,39] databases, which includes both novel phosphopeptides in proteins that are, so far, not annotated as phosphoproteins, as well as novel phosphopeptides in other regions of known phosphoproteins (see Table 1 and S2). Note that Table 1 lists only the novel phosphopeptide with a high-confidence pRS score cutoff (>30); a longer list of all potential phosphopeptides is shown in Table S2. Additionally, Table S3 (a modified version of Table S2) links the identified phosphopeptides and the associated phosphoproteins. Inspection of these tables also reveals a progressive increase in the number of phosphopeptides associated with a particular (phospho)protein from the crude extract to the final PAPE fraction. Examples include RD29A (desiccation-responsive protein 29; also known as low-temperature-responsive protein 78, At5g52310), NR2 (nitrate reductase 2, At1g37130) and two proteins with tetratricopeptide repeat (TPR) domains (At1g01320 and At4g28080) (Figure 4). Taken together, these phosphopeptide detection data demonstrate the efficacy of the PAPE procedure to identify (novel) phosphoproteins.

**Table 1:** List of novel phosphopeptides identified in this study (q-value < 0.05; pRS score > 30), which are not found in the P3DB or PhosPhAt 3.0 phosphoprotein databases.

	No.	Protein code	Description	Sequence	MH+[Da]	q-Value	PEP	pRS Score	# PSMs	pRS Site Probabilities
**Crude extract**	1	AT1G14010.1	emp24/gp25L/p24 family/GOLD family protein	SSIVLLILSILSPVTLSIR###	2,184.20708	0.016281	0.3789499	58	11	S(1): 15.3; S(2): 15.3; S(9): 84.3; S(12): 84.6; T(15): 0.5; S(17): 0.0
	2	AT2G40840.1	disproportionating enzyme 2	VEKPLGVFMNKSDQDDSVVVQFK	2,689.27768	0.021343	0.6031149	33	1	S(12): 0.4; S(17): 99.6
	3	AT2G38280.1	AMP deaminase, putative/myoadenylate deaminase, putative	SNGHVYVDEIPPGLPRLHTPSEGRASVHGASSIR	3,832.73672	0.022388	0.6381906	32	2	S(1): 33.1; Y(6): 33.1; T(19): 33.1; S(21): 4.1; S(26): 95.3; S(31): 50.7; S(32): 50.7
	4	AT4G38740.1; AT2G21130.1	rotamase CYP 1/ Cyclophilin-like peptidyl-prolyl cis-trans isomerase family protein	HTGPGILSMANAGANTNGSQFFICTVK	2,873.30117	0.027543	0.4591594	37	3	T(2): 0.7; S(8): 95.1; T(16): 2.0; S(19): 2.0; T(25): 0.1
	5	AT4G23670.1	polyketide cyclase/dehydrase and lipid transport superfamily protein	ATSGTYVTEVPLKGSAEK###	1,917.91213	0.032722	0.4923883	48	4	T(2): 24.5; S(3): 24.5; T(5): 24.5; Y(6): 24.5; T(8): 1.9; S(15): 0.0
	6	AT1G70200.1	RNA-binding (RRM/RBD/RNP motifs) family protein	QFTGQSLAFGKVIKQIK	1,973.05167	0.047422	0.6522376	35	12	T(3): 86.5; S(6): 13.5
**40% AS**	1	AT5G56740.1	histone acetyltransferase of the GNAT family 2	LSQILVLPSFQGK	1,509.80133	0.011279	0.2471017	30	4	S(2): 0.4; S(9): 99.6
	2	AT1G23740.1	oxidoreductase, zinc-binding dehydrogenase family protein	NAALATTTATTPVLRR	1,736.90842	0.015443	0.4098554	40	1	T(6): 12.5; T(7): 12.5; T(8): 59.5; T(10): 12.5; T(11): 3.0
	3	AT5G52790.1	CBS domain-containing protein with a domain of unknown function (DUF21)	LLDLLLGKRHSTLLGR###	1,885.07854	0.023892	0.3993647	51	11	S(11): 1.7; T(12): 98.3
	4	AT4G28000.1	P-loop containing nucleoside triphosphate hydrolases superfamily protein	HTRNLAPGSK	1,160.55168	0.03559	0.754117	50	3	T(2): 0.0; S(9): 100.0
	5	AT1G72150.1	PATELLIN 1	SSFVFVSDFRNAPGLGKR	2,064.01040	0.039425	0.6264254	39	1	S(1): 1.0; S(2): 1.0; S(7): 98.0
	6	AT2G04842.1	threonyl-tRNA synthetase, putative/threonine-tRNA ligase, putative	SRFGGELGTIPVDDLINKINIAVETR###	3,067.42545	0.041246	0.6307821	38	1	S(1): 100.0; T(9): 100.0; T(25): 100.0
	7	AT3G22760.1	tesmin/TSO1-like CXC domain-containing protein	VIRNSDSIIEVGEDASK###	1,911.89481	0.042639	0.8216446	52	1	S(5): 0.0; S(7): 0.1; S(16): 99.9
	8	AT3G16950.1; AT4G16155.1	lipoamide dehydrogenase 1/ dihydrolipoyl dehydrogenases	DIIIATGSVPFVPK	1,536.80143	0.043742	0.5158506	38	2	T(6): 12.3; S(8): 87.7
**Crude extract + MOAC**	1	AT1G56220.4	dormancy/auxin associated family protein	HHTFSFRPSSGNDQSEAGSAR###	2,354.98525	0	7.2906E-05	36	16	T(3): 13.7; S(5): 57.9; S(9): 13.7; S(10): 13.7; S(15): 1.1; S(19): 0.1
	2	AT2G17410.2	ARID/BRIGHT DNA-binding domain-containing protein	HSEENQSPHHHANNVMEQDQAAEER	3,004.19179	0	0.00011139	60	9	S(2): 97.1; S(7): 2.9
	3	AT5G52310.1	low-temperature-responsive protein 78 (LTI78)	MDQTEEPPLNTHQQHPEEVEHHENGATK	3,342.38857	0	2.1345E-05	36	16	T(4): 96.1; T(11): 3.8; T(27): 0.0
	4	AT5G55160.1	small ubiquitin-like modifier 2	SATPEEDKKPDQGAHINLK###	2,237.97500	0.000487	0.00798287	31	2	S(1): 100.0; T(3): 100.0
	5	AT2G24270.2	aldehyde dehydrogenase 11A3	AGTGLFAEILDGEVYK###	1,762.82077	0.000503	0.0115286	38	3	T(3): 100.0; Y(15): 0.0
	6	AT1G45207.2	remorin family protein	GWSSERVPLR	1,266.59882	0.00075	0.01489954	38	24	S(3): 50.0; S(4): 50.0
	7	AT1G01100.1; AT5G47700.1	60S acidic ribosomal protein family	STVGELACSYAVMILEDEGIAITADK	2,836.31522	0.000976	0.01672018	48	4	S(1): 25.0; T(2): 25.0; S(9): 25.0; Y(10): 25.0; T(23): 0.0
	8	AT4G12420.1	cupredoxin superfamily protein	RPLTGPAKVATSIINGTYR	2,175.06754	0.002939	0.08369295	36	3	T(4): 99.1; T(11): 7.4; S(12): 91.8; T(17): 0.9; Y(18): 0.9
	9	AT1G74920.1; AT3G48170.1	aldehyde dehydrogenase 10A8/9	SPLIVFDDVDLDK	1,555.73259	0.009502	0.2584146	70	2	S(1): 100.0
	10	AT3G28710.1	ATPase, V0/A0 complex, subunit C/D	AVNITINSIGTELTR###	1,681.86216	0.015828	0.3155473	37	30	T(5): 0.0; S(8): 77.3; T(11): 11.3; T(14): 11.3
	11	AT1G73610.1	GDSL-like Lipase/Acylhydrolase superfamily protein	SYETIAPQIIENIKAK###	1,977.93410	0.017056	0.3870838	30	18	S(1): 50.3; Y(2): 50.3; T(4): 99.3
	12	AT2G41110.1	calmodulin 2	ADQLTDDQISEFK	1,589.66288	0.019757	0.513722	50	5	T(5): 100.0; S(10): 0.0
	13	AT1G70200.1	RNA-binding (RRM/RBD/RNP motifs) family protein	QFTGQSLAFGKVIKQIK	1,973.05405	0.02041	0.4607051	40	7	T(3): 50.0; S(6): 50.0
	14	AT2G22400.1	S-adenosyl-L-methionine-dependent methyltransferases superfamily protein	EIRKNQTLER	1,366.68921	0.02499	0.5231273	38	1	T(7): 100.0
	15	AT4G30630.1	unknown protein	LSESGGLEVPRKPSGERK###	2,006.01230	0.031388	0.6183366	32	1	S(2): 0.1; S(4): 0.2; S(14): 99.7
	16	AT5G64090.1	unknown protein	ASHDLNPQAILATR	1,586.76555	0.035	0.6582299	57	1	S(2): 0.0; T(13): 100.0
	17	AT1G80380.3	P-loop containing nucleoside triphosphate hydrolases superfamily protein	GNAGSHDLKLSVETLEALSKLTK###	2,491.28416	0.043032	0.7887968	36	1	S(5): 0.2; S(11): 0.1; T(14): 0.2; S(19): 9.4; T(22): 90.1
**40% AS + MOAC (PAPE)**	1	AT5G52310.1	low-temperature-responsive protein 78 (LTI78)	SHELDLKNESDIDKDVPTGFDGEPDFLAK	3,311.49355	0	0.0027675	58	8	S(1): 0.6; S(10): 98.8; T(18): 0.6
	2	AT1G01320.2	tetratricopeptide repeat (TPR)-like superfamily protein	STQPSSGNAKTAGETSEEDGLKTDASSVEPPTLSSTVQSEAYHTK###	4,690.11245	0.000811	0.04504684	41	10	S(1): 2.7; T(2): 2.7; S(5): 2.7; S(6): 2.7; T(11): 2.7; T(15): 17.1; S(16): 17.1; T(23): 17.1; S(26): 17.1; S(27): 17.1; T(32): 0.5; S(34): 0.1; S(35): 0.1; T(36): 0.1; S(39): 0.0; Y(42): 0.0; T(44): 0.0
	3	AT3G11130.1; AT3G08530.1	clathrin, heavy chain	EYSGKVDELIK###	1,360.63437	0.000811	0.04561926	49	6	Y(2): 99.7; S(3): 0.3
	4	ATMG00285.1	NADH dehydrogenase 2A	KSEFSTEAGSK###	1,250.52198	0.001809	0.09635145	66	3	S(2): 0.0; S(5): 9.0; T(6): 91.0; S(10): 0.0
	5	AT1G20620.1	catalase 3	MDPYKYRPSSAYNAPFYTTNGGAPVSNNISSLTIGER	4,118.89516	0.002241	0.04873965	47	14	Y(4): 2.0; Y(6): 2.0; S(9): 15.6; S(10): 15.6; Y(12): 15.6; Y(17): 15.6; T(18): 15.6; T(19): 15.6; S(26): 2.0; S(30): 0.3; S(31): 0.1; T(33): 0.0
	6	AT3G18780.2	actin 2	AEADDIQPIVCDNGTGMVKAGFAGDDAPR###	3,070.31753	0.00444	0.09388046	45	2	T(15): 100.0
	7	AT5G09810.1; AT2G37620.1	actin 1/7	ADGEDIQPLVCDNGTGMVKAGFAGDDAPR###	3,056.32534	0.005154	0.1148702	36	5	T(15): 100.0
	8	AT5G56180.1	actin-related protein 8	TVVLTGGSACLPGLSER###	1,796.85564	0.005762	0.1187534	68	2	T(1): 0.0; T(5): 0.0; S(8): 0.1; S(15): 99.8
	9	AT1G49240.1	actin 8	ADADDIQPIVCDNGTGMVKAGFAGDDAPR###	3,056.32534	0.008319	0.1784335	36	3	T(15): 100.0
	10	AT3G02830.1	zinc finger protein 1	NKAGIAGRVSLNMLGYPLR	2,110.10227	0.016331	0.3097203	47	1	S(10): 100.0; Y(16): 0.0
	11	AT1G64790.1	ILITYHIA	SPIVSAAAFENLVK	1,525.75934	0.017784	0.3293382	48	5	S(1): 10.3; S(5): 89.7
	12	AT4G38770.1	proline-rich protein 4	KEVPPPVPVYKPPPK###	1,751.95328	0.026158	0.4063287	34	1	Y(10): 100.0
	13	AT4G31120.2	SHK1 binding protein 1	DVHLGIEPTTATPNMFSW###	2,095.92093	0.031227	0.430492	31	1	T(9): 0.5; T(10): 0.5; T(12): 1.4; S(17): 97.6
	14	AT5G16330.1	NC domain-containing protein-related	RGTCTIAPSDPCDEVISR###	2,193.86269	0.039343	0.684857	64	3	T(3): 5.3; T(5): 0.4; S(9): 94.3; S(17): 100.0

###: Peptides marked with “###” are from proteins that are, so far, not annotated as phosphoproteins. All other peptides belong to known phosphoproteins, but are themselves not reported as being phosphorylated. Abbreviations: MH+ = Positive ion mode pseudo-molecular ion; Da = Dalton; PEP = Posterior error probability; PSM = peptide spectrum match.

However, there are also cases where no phosphopeptides could be identified for the putative phosphoprotein enriched in the PAPE fraction (e.g., MPK4 or MPK6). This is possibly one of the caveats of the present study, which is that when compared to the reproducibility in identification of the (putative) phosphoprotein (see Table S1, Figure 2B), there is often difficulties or variation in the phosphopeptide identification between replicate measurements. Contrary to expectation, the absolute number of phosphopeptide identified is not particularly high, despite the increased phosphoprotein detection (Figure 3C). However, such limitations can be attributed to the fact that the subsequent tryptic digestion reintroduced a complex peptide mixture, thereby hindering the phosphopeptide identification by MS as a consequence of the over-representation of non-phosphorylated peptides over phosphopeptides [40]. It is known that phosphoprotein enrichment procedures will increase the number of phosphorylated proteins, but this does not necessarily translate to larger numbers of identified phosphorylated peptides [9]. For this purpose, an additional phosphopeptide enrichment step to the current PAPE procedure may be included to enhance phosphopeptide identification. However, due to the different efficiencies in capturing mono-phosphorylated and multiple phosphorylated peptides from complex peptide mixtures [41], this was not done in the current study to avoid losing the identification of certain phosphoproteins. The current PAPE procedure is mainly designed to detect phosphoproteins from green plant tissues.

## 4. Conclusions

We report here that a simple ammonium sulfate fractionation step can be used to eliminate abundant RuBisCO proteins and simultaneously enrich phosphoproteins from *Arabidopsis* leaves. A combination of this step with MOAC phosphoprotein enrichment, which we termed PAPE, enabled the identification of low abundance phosphoproteins, including several that are not annotated in the P3DB and PhosPhAt 3.0 databases. Overall, the PAPE procedure performed better than MOAC alone to enrich phosphoproteins. While some proteins will be missed by the PAPE procedure, because of removal during the ammonium sulfate precipitation step, the Pro-Q Diamond phosphostain indicated that the bulk of phosphoproteins are actually within the fraction used for analysis (see Figure 1B). Thus, by eliminating RuBisCO and enriching phosphoproteins, the PAPE procedure reduces the effective dynamic range of protein abundance in the plant proteome and ameliorates the detection of phosphoproteins. Its facile handling allows it to be implemented in any laboratory. We also envisage that the inclusion of a phosphopeptide enrichment step to the current PAPE fraction would further improve the mapping of the plant phosphoproteome.

## Supplementary Materials

Supplementary File 1

Supplementary File 2

Supplementary File 3
